# HIV-1 uncoating in human CD4^+^ T cells: kinetic and functional analyses

**DOI:** 10.1186/1742-4690-10-S1-P13

**Published:** 2013-09-19

**Authors:** Nan-Yu Chen, Lihong Zhou, Paul G Gane, Amanda Price, Madeleine Zufferey, Jeremy Luban, Leo James, David Selwood, Ariberto Fassati

**Affiliations:** 1 Wohl Virion Centre, Division of Infection & Immunity, London, UK; 2 Medicinal Chemistry Group, UCL, London, UK; 3 MRC Laboratory for Molecular Biology, Cambridge, UK; 4 Center for AIDS Research, Massachusetts, USA

## Background

After HIV-1 fuses with the host cell, the viral core is released into the cytoplasm and starts shedding the capsid proteins (CA), a process called uncoating. Uncoating impacts on reverse transcription, nuclear import and integration; however, it is poorly understood.

## Materials and methods

We have developed an uncoating assay using human CD4+ T that stably express the restriction factor TRIMCyp. TRIMCyp targets the HIV-1 capsid core to block infection, however it can be specifically competed by Cyclosporine A (CsA). By washing out CsA at different time points in TRIMCyp expressing cells, we were able to determine for how long after infection TRIMCyp can target the core. We then evaluated the effect on uncoating of the drug Coumermycin-A1, which targets HIV-1 capsid and blocks integration, and the effect of Nup153, which promotes HIV-1 nuclear import and integration.

## Results

In our experimental system, HIV-1 uncoating occurred about 100-120 min after infection. Coumermycin-A1 accelerated uncoating significantly and the effect was capsid-dependent because the drug did not change the kinetics of uncoating of two capsid point mutants, N74D and A105S. Faster uncoating did not affect reverse transcription. Experiments using the NNRTI Nevirapine indicated that an initial uncoating step precedes reverse transcription but viral DNA synthesis helps uncoating at a later stage, at least in a subset of cores. Our assay revealed a degree of heterogeneity in the kinetics of uncoating whereby most cores did not need reverse transcription to uncoat but a subset did. Faster uncoating induced by Coumermycin-A1 prevented accumulation of even small amounts of HIV-1 capsid into the nucleus of infected cells and inhibited integration. Depletion by shRNA of nucleoporin Nup153, which is located at the nuclear basket of the nuclear pore complex, appeared to accelerate uncoating in CD4+ T cells, suggesting that this nucleoporin might stabilize the core.

## Conclusions

We have determined kinetics of HIV-1 uncoating in human CD4+ T cells. We found that there is a heterogeneous population of cores that are more or less dependent on reverse transcription for uncoating. Coumermycin-A1 appears to block HIV-1 integration by accelerating uncoating and preventing accumulation of capsid into the nucleus. Nup153 appears to stabilise the core. Taken together, these results suggest that small number of capsid proteins are necessary for efficient integration and that a final uncoating step may take place in the nucleus of infected cells.

